# Number of siblings and the risk of solid tumours: a nation-wide study

**DOI:** 10.1038/sj.bjc.6603760

**Published:** 2007-04-24

**Authors:** A Altieri, K Hemminki

**Affiliations:** 1 Division of Molecular Genetic Epidemiology, German Cancer Research Centre (DKFZ), Im Neuenheimer Feld 580, D-69120 Heidelberg, Germany; 2 Center for Family Medicine, Karolinska Institute, Alfred Nobels Allé 12, SE 14183 Huddinge, Sweden

**Keywords:** birth order, sibship size, number of sibling, cohort study, cancer risk

## Abstract

We analysed the effects of number of siblings on the risk of solid tumours using the Swedish Family-Cancer Database, including population-based information on over 11 million individuals and more than 178 000 cancer patients diagnosed between 1958 and 2004. Incidence rate ratios (RRs), estimated by Poisson regression models, were adjusted for age, sex, birth cohort, area of residence and socioeconomic status. Having eight or more siblings *vs* none increased the risk of stomach cancer (RR=1.83, 95% confidence interval (CI), 1.44–2.34). Anal cancer diagnosed before age 40 showed the strongest association with the total siblings (RR=3.27, 95% CI, 2.04–5.26 for five or more siblings *vs* none). Endometrial (RR=0.76, 95% CI, 0.70–0.82), testicular (RR=0.71, 95% CI, 0.62–0.82), skin cancer (RR=0.82, 95% CI, 0.69–0.97) and melanoma (RR=0.72, 95% CI, 0.65–0.79) showed strong decreased risks for five or more siblings *vs* none. Prostate cancer risk for those with five or more older siblings *vs* none was 1.38 (95% CI, 1.23–1.55). Having five or more younger siblings was most strongly associated with stomach cancer (RR=1.59, 95% CI, 1.29–1.95) and melanoma (RR=0.68, 95% CI, 0.59–0.79). We conclude that sibship characteristics are strong correlates of cancer risk at several sites; plausible interpretations include socioeconomic status.

Sibship size and birth order are not *per se* explanatory variables for cancers, but their biological and socioeconomic correlates may be. For example, birth order has been associated with birth weight, a risk factor for breast cancer (Juntunen *et al*, 1997; Potischman and Troisi, 1999; Barba *et al*, 2006). *In utero* estradiol levels decrease with birth order, with potential implications for risks of breast, testicular and possibly other cancer sites (Bernstein *et al*, 1986; Panagiotopoulou *et al*, 1990; Trichopoulos, 1990; Hsieh *et al*, 1991; Prener *et al*, 1992; Westergaard *et al*, 1998; Potischman and Troisi, 1999; Petridou *et al*, 2000; Weir *et al*, 2000; Hodgson *et al*, 2004; Richiardi *et al*, 2004; Sorensen *et al*, 2005). Family crowding necessarily involves intimate contacts between its members, with potential effects on infectious diseases (Kinlen *et al*, 1990; Altieri *et al*, 2006a, 2006b). At least until a few decades ago, sibship size correlated with various socioeconomic and dietary factors (La Vecchia *et al*, 1995; Kivi *et al*, 2005; Garg *et al*, 2006). Total number of siblings may reflect genetic risk factors, as early-onset cancers or other inherited diseases may limit the parental reproductive period and show higher risks for small families because of selection.

We investigate here the effects of the number of siblings on the risk of common solid tumours using data from the Swedish Family-Cancer Database, the subject of only one previous study (Hemminki and Mutanen, 2001). Effects of sibship size on lymphoproliferative and nervous system malignancies have been reported elsewhere (Altieri *et al*, 2006a, 2006b). The present study includes over five times as many cases as previous analyses, more cancer sites, and an older cohort age, thereby providing more robust estimates of associations, including differences between younger and older siblings by age and gender, which provide further insights into their timing and mechanisms.

## MATERIALS AND METHODS

The Swedish Family-Cancer Database was created in the mid-1990s by linking census information, death notifications and the administrative sibship register at Statistics Sweden to the Swedish Cancer Registry (Hemminki *et al*, 2001). It includes persons born in Sweden after 1931 with their biologic parents, totalling over 11 million individuals, each with a unique technical identification number, allowing construction of families. Neoplasms were retrieved from the Swedish Cancer Registry from 1958 to 2004. This Registry is based on statutory reports of cancer cases from clinicians and others and is considered to be now almost 100% complete (The National Board of Health and Welfare, 2002). Pathologists or cytologists report every cancer case diagnosed on operative specimens, biopsies, cytological specimens, bone marrow aspirates and autopsies. The incidence of tumours according to the Database has been validated previously (Hemminki *et al*, 2001). Data on parity were complete, and data on socioeconomic index and area of residence were based on population censuses of Statistics Sweden from the years 1960, 1970, 1980 and 1990. Overall, the analyses covered 178 365 individuals with a cancer diagnoses.

Four-digit diagnostic codes from the seventh revision of the International Statistical Classification of Diseases (ICD-7) and subsequent ICD classifications are available. Cancer site groupings were upper aerodigestive tract (140–141, 143–148, 150, 161), liver and gallbladder (155–156) and lung (162–163), covering the period 1958–2004. The age of parents was not restricted, but the maximum age of offspring was 72 years.

Variation in distribution among different socioeconomic classes only occurred in families with one child (professional 18%, agricultural and forestry 14.5%, other manual workers 25%, self-employed 15%, other 27.5%) and in families with five or more children (professional 7%, agricultural and forestry 15.3%, other manual workers 23.6%, self-employed 14%, other 40.1%). Families with five or more children decreased from 14% before the 1960s to 4.5% in the 1990s and thereafter. The decrease in the proportion for families with four children was considerably lower (from 14 to 8%). The number of families with 2–3 children varied between 30 and 40% in different calendar periods. Families with one child decreased from 16 to 7% before the 1990s and increased to 15% thereafter.

The number of older siblings corresponds to birth order. For example, individuals with no older siblings (singletons and first borns) correspond to birth order one, and those with one older sibling equal birth order two. The number of older siblings can be seen as parity for the mother at the index pregnancy. For each child there are data on both parents at the time of birth. In the case of divorce, we were not able to verify which children remained with the same sibship. However, we assumed that all children lived with the mother.

### Statistical methods

Follow-up was started on the date of birth, the date of immigration, or on 1 January 1958, whichever occurred last. Follow-up ended on the date of diagnosis of the first primary neoplasm, date of death, date of emigration or the closing date of the study, 31 December 2004, whichever occurred first.

Person-years and cancer cases were counted and grouped by the study explanatory variables (sex, age, birth cohort, total number of siblings, number of older and younger siblings, socioeconomic status and area of residence) during the follow-up period for the child. The Poisson regression models (multiplicative model and logarithm of person-years as offset) was applied to the data and the GENMOD-procedure of the SAS-system V.9.1 was used. The term rate ratio (RR) was used for the exp (*β*), where *β* is the estimated model parameter value; this was interpreted as an incidence rate ratio (e.g. RR is the incidence rate ratio for sibship size 2 as compared to sibship size 1 as the reference category).

The main explanatory variables were total number of siblings, number of older and younger siblings. Other covariates included in the statistical models were age at diagnosis (*quinquennia*, from 0 to 72), year of birth (birth cohort, four categories: <1970, 1970–1979, 1980–1989, ⩾1990), socioeconomic status (four categories: professional, agricultural and forestry, other manual workers, self-employed and others) and area of residence (five categories: Stockholm area, Göteborg-Malmö area, the two largest cities in southern Sweden; Götaland, Svealand and Norrland). Other variables that were tested, but not included in the models were parity (no child, 1–2, 3–4, 5 or more children), age at first parturition (no child, <=20, 21–29, 30–39, ⩾40 years of age) and family history of cancer in first-degree relatives. For children and for each individual with missing data on socioeconomic status we used the information of the parents. Thus, socioeconomic information was available from at least one census for 94% of the population.

## RESULTS

Table 1 shows the effect of total number of siblings on 22 different cancer sites or groups. Compared to singletons, individuals with five or more siblings had an increased risk of stomach (RR=1.48), lung (RR=1.13) and cervical (RR=1.19) cancers, each with a significant trend in risk (*P*<0.001). The risk of stomach cancer for eight or more siblings was 1.83 (95% confidence intervals (CI), 1.44–2.34, *P* trend <0.001) based on 60 cases (data not shown). The testis (RR=0.71), melanoma (RR=0.72), endometrium (RR=0.76) and skin (RR=0.82) showed significant decreased risks for five or more siblings compared to none, with significant trends in decreasing risk (*P*<0.001). The risk of testicular cancer for eight or more siblings was 0.58 (95% CI, 0.38–0.90, *P* trend <0.001, data not shown).

Table 2 gives the risks for total number of older siblings for anal and cervical cancer according to age at diagnosis. For age at diagnosis <40 years of anal cancer, the RR for five or more siblings compared to none was 3.27 (*P* trend <0.001). Test for heterogeneity for age in total number of siblings was significant (*P* trend <0.01). No significant association was found for diagnoses over 40 years of age. No clear pattern of association was found for number of siblings in strata of sex. Having five or more siblings increased the risk of cervical cancer diagnosed before age 40 years (RR=1.16).

When we estimated the risk for number of younger or older siblings, having five or more older siblings compared to none increased the risk of prostate cancer (RR=1.38, 95% CI, 1.23–1.55, *P* trend <0.001). Strong inverse associations were found for five or more older siblings compared to none for endometrial (RR=0.60, 95% CI, 0.46–0.78, *P* trend =0.001) and testicular (0.70, 95% CI, 0.56–0.89, *P* trend=0.001) cancer, and melanoma (RR=0.67, 95% CI, 0.55–0.80, *P* trend<0.001). Having five or more younger siblings compared to none was associated with an increased risk of stomach (RR=1.59, 95% CI, 1.29–1.95, *P* trend <0.001), and kidney (RR=1.17, 95% CI, 1.02–1.35, *P* trend <0.001) cancers, and decreased the risk of endometrial (0.78, 95% CI, 0.70–0.87, *P* trend <0.001), ovary (RR=0.87, 95% CI, 0.77–0.99, *P*=0.006), prostate (RR=0.78, 95% CI, 0.74–0.82, *P* trend<0.001) and testicular (RR=0.79, 95% CI, 0.63–0.98, *P* trend<0.001) cancers, and melanoma (RR=0.68, 95% CI, 0.59–0.79, *P* trend <0.001). Analyses in strata of age showed no clear patterns of risks for number of younger or older siblings.

Seminomas were significantly associated with four or more siblings (RR=0.74, 95% CI, 0.66–0.83), while no significant association was found for non-seminomas (RR=0.90, 95% CI, 0.81–1.08, data not shown). The risk for three or more older siblings compared to none was 0.85 (95% CI, 0.77–0.98) for seminomas and 0.86 (95% CI, 0.73–1.01) for non-seminomas. The risk for three or more younger siblings compared to none was 0.81 (95% CI, 0.75–0.90) for seminomas and 0.75 (95% CI, 0.69–0.89) for non-seminomas.

The patterns of risks of the other cancer sites or groups were homogeneous across strata of age at diagnosis and sex. The risk by gender of siblings did not materially change for number of older sisters and number of older brothers. The analyses that considered average age distance between siblings showed no clear patterns.

## DISCUSSION

Our results show that the number of siblings correlates with cancer risk at different sites. Subjects coming from large families showed a higher risk of stomach cancer. The association was significant for families of four or more offspring, and was increased up to approximately twofold for eight or more siblings, in agreement with at least two case–control studies from Italy and Sweden (Hansson *et al*, 1994; La Vecchia *et al*, 1995). The association between stomach cancer and family size was not different in strata of age, sex, period and socioeconomic status. *Helicobacter pylori* is the strongest determinant of gastric cancer development, and an early acquisition of infection has been reported to be directly associated with domestic crowding and sibship size (Mendall *et al*, 1992; Webb *et al*, 1994; Blaser *et al*, 1995; Koch *et al*, 2005). Stomach cancer has been associated with low socioeconomic status and unfavourable living conditions, such as childhood deprivation (Logan, 1982; Howson *et al*, 1986). Larger families were probably a stronger indicator of poor living conditions in the early part of the last century than more recently. However, in our dataset the patterns of risk were not heterogeneous in strata of calendar periods and socioeconomic index.

One interesting finding of our study is the threefold increased risk of anal cancer for age at diagnosis before 40 years. For cervical cancer, we observed a 16% increased risk for large sibship size in the same age group. Specific types of human papillomavirus have been identified as causative agents of at least 90% of cancers of the cervix and more than 50% of other anogenital cancers (zur Hausen, 1996; Hernandez *et al*, 2005). Vertical transmission from mother to infant during birth is well established (zur Hausen and de Villiers, 2005), while postnatal acquisition by nonsexual horizontal transmission can occur rarely (Frega *et al*, 2003; Sinclair *et al*, 2005).

Lung cancer was more common in larger families. This association has never been reported before and it can be possibly explained by a residual confounding effect of socioeconomic factors (Hemminki and Chen, 2006).

We found no substantial increased risk for breast cancer with increasing the number of siblings. The risks were not modified by the women's own reproductive history and age at diagnosis. These data are not consistent with the epidemiological evidence that birth weight increases with birth order, and the correlation of birth weight and breast cancer risk (Trichopoulos, 1990; Michels *et al*, 1996; Sanderson *et al*, 1996; Juntunen *et al*, 1997; Andersson *et al*, 2000; Kaijser *et al*, 2000). No association was found between the number of siblings and male breast cancer (Petridou *et al*, 2000; Sorensen *et al*, 2005). The strong inverse association between the number of siblings and the risk of endometrial cancer, consistent across strata of age, has never been reported before. Endometrial cancer is probably the most oestrogen-dependent neoplasm, and our results suggest that the oestrogen *in utero* exposure hypothesis may be valid not only for testicular but also for endometrial cancer.

Early-onset cancers or other inherited diseases may limit the reproductive period of the parents and show higher risks for small families because of selection (Hemminki and Chen, 2006). Testicular cancer consistently showed the linear inverse associations with the number of siblings. These results are in broad agreement with a nested case–control study from Sweden based on some 3000 cases retrieved from the Cancer Registry (Richiardi *et al*, 2004). The decreased risk observed for number of older siblings shows that individuals of late birth order are at lower risk of testicular cancer. This finding is consistent with the *in utero* exposure hypothesis that firstborns, exposed to higher prenatal oestrogen levels compared to later ones, run a higher risk of testicular cancer (Westergaard *et al*, 1998; Richiardi *et al*, 2002), as confirmed by other measures of prenatal exposures to female hormones, such as maternal use of exogenous hormones during pregnancy (Weir *et al*, 2000). We observed no material difference between seminomas and non-seminomas, histopathological subtypes of testicular cancer (Sabroe and Olsen, 1998).

For melanoma, the number of older or younger siblings showed a protective effect. In this population melanoma has been found to have a strong association with socioeconomic index (Hemminki *et al*, 2003). The most plausible explanation is that in large families the affordability of sun holidays in exotic countries and other high social class behaviours at risk, including use of solarium, is less than in small families (Bentham and Aase, 1996; Hemminki and Mutanen, 2001; Hemminki and Li, 2004).

The total number of siblings was not associated with prostate cancer risk. We observed a 38% increased risk of prostate cancer for men having five or more older siblings. This could be explained by a surveillance bias, since men with an older brother, particularly with an older brother affected with cancer, are more likely to undergo a medical examination or to participate in a screening programme, as already reported from our Database and other sources (Hsieh *et al*, 1999; Beebe-Dimmer *et al*, 2004; Bermejo and Hemminki, 2005).

In the present study, all malignancies were based on nationwide registered sibship structures and medical diagnoses with histopathologic confirmation, minimising risks for recall biases and loss to follow-up. The ascertainment of relatives was complete, giving further reassurance. A potential effect of sibship size and birth order on cancer risk may be evident only for subjects with a sibship history of cancer, showing that environmental influences may be overwhelmed by genetic predisposition to cancer. However, in our multivariate models we included a term for sibship history of cancer, and for other potential confounding factors. Further, due to the low number of familial cases, analyses repeated excluding individuals with a family history of cancer lead to similar results. The major weakness of our study is the lack of availability of more direct markers of environmental exposures, including smoking habits, number and type of infections, females' hormones, anthropometric measures and serological data. The availability of such data from at least a subset of individuals of our population could add further evidence to the proposed hypotheses. However, they are not likely to confound or modify the effect of sibship size or the number of siblings. A possible concern about these results is the confounding family size appeared to be different across socioeconomic class in families with one child or for five or more children. However, even for cancer sites like stomach, cervix, lung, melanoma and testis that show associations with socioeconomic factors in this Database (Hemminki *et al*, 2003), the risk estimates for family size were not significantly different across socioeconomic status. Thus, a residual social effect for these cancer sites cannot be ruled out. Family size varied across calendar period. However, the association of family size and cancer risk were not significantly different across strata of birth cohorts.

The current investigation represents the only population-based study providing reliable systematic quantifications of the effects of birth order and sibship size on the risk of solid tumours. We conclude that sibship characteristics are strong correlates of cancer risk at several sites with several possible interpretations. Given the strong correlations of the number of siblings and risks for specific cancers, some of which are new, further investigations of more direct markers of exposure are warranted.

## Figures and Tables

**Table 1:** Incidence rate ratios (RR)^a^ for the total number of siblings

	**Total number of siblings**															
	**None^b^**			**1–2**				**3–4**				**5 or more**				
**Site**	**Obs**	**RR**	**Obs**	**RR**	**95%**	**CI**	**Obs**	**RR**	**95%**	**CI**	**Obs**	**RR**	**95%**	**CI**	**Total**	***P*-trend**
Upper aerodigestive tract	1190	1.00	3235	1.01	0.90	1.12	1141	1.00	0.88	1.14	375	0.89	0.74	1.07	5941	0.981
Stomach	622	1.00	1649	1.01	0.93	1.10	667	1.14	1.03	1.26	324	1.48	1.31	1.67	3262	<0.001 (+)
Colon	2200	1.00	5734	0.95	0.89	1.02	1909	0.93	0.85	1.01	721	0.98	0.88	1.10	10564	0.324
Rectum	1332	1.00	3247	0.98	0.91	1.05	1153	0.99	0.91	1.07	422	0.98	0.87	1.10	6154	0.994
Anus	90	1.00	283	1.16	0.94	1.44	96	1.13	0.87	1.46	40	1.28	0.92	1.80	509	0.150
Pancreas	672	1.00	1622	0.97	0.90	1.05	597	1.01	0.92	1.11	246	1.11	0.98	1.26	3137	0.021
Lung	2315	1.00	5883	1.04	0.98	1.10	2340	1.14	1.06	1.23	891	1.13	1.02	1.25	11429	<0.001 (+)
Breast	8309	1.00	24520	1.04	1.02	1.07	8037	1.02	0.99	1.04	2650	0.96	0.93	1.00	43516	<0.001 (+)
Cervix	989	1.00	3754	1.03	0.97	1.10	1386	1.17	1.09	1.26	461	1.19	1.08	1.31	6590	<0.001 (+)
Endometrium	1497	1.00	3090	0.88	0.84	0.92	1086	0.87	0.83	0.93	361	0.76	0.70	0.82	6034	<0.001 (−)
Ovary	1299	1.00	3861	1.00	0.95	1.05	1248	0.93	0.88	0.99	466	0.98	0.90	1.06	6874	0.257
Prostate	4755	1.00	10164	1.06	1.02	1.10	3447	1.06	1.02	1.11	1155	0.97	0.91	1.03	19521	0.301
Testis	732	1.00	3569	0.94	0.87	1.01	898	0.84	0.76	0.92	208	0.71	0.62	0.82	5407	<0.001 (−)
Kidney	1003	1.00	2940	0.98	0.89	1.07	1034	0.99	0.88	1.11	377	1.00	0.86	1.16	5354	0.493
Urinary bladder	1501	1.00	3698	0.98	0.91	1.06	1308	1.00	0.91	1.09	489	1.01	0.89	1.15	6996	0.717
Melanoma	2772	1.00	9864	1.02	0.97	1.07	2711	0.89	0.84	0.94	692	0.72	0.65	0.79	16039	<0.001 (−)
Skin	900	1.00	2386	0.99	0.90	1.09	683	0.84	0.75	0.95	235	0.82	0.69	0.97	4204	0.001 (−)
Thyroid	561	1.00	2273	1.05	0.95	1.16	747	1.09	0.97	1.22	226	1.04	0.88	1.22	3807	0.410
Endocrine glands	1011	1.00	3450	1.03	0.95	1.13	1175	1.06	0.95	1.18	382	1.02	0.88	1.18	6018	0.506
Bone	192	1.00	985	0.95	0.81	1.12	283	0.97	0.80	1.18	67	0.85	0.63	1.13	1527	0.485
Connective tissue	415	1.00	1499	0.88	0.77	0.99	461	0.87	0.75	1.01	150	0.90	0.73	1.12	2525	0.243
Liver and gallbladder	620	1.00	1562	0.95	0.87	1.03	552	0.94	0.84	1.05	223	1.01	0.88	1.17	2957	0.411

a Estimated by Poisson regression models including terms for age, sex, period, county and socioeconomic index.

b Reference category.

**Table 2:** Incidence rate ratios (RR)^a^ for anal and cervical cancer for the total number of siblings according to age at diagnosis

	**None^b^**			**1 or 2**				**3 or 4**				**5 or more**				
	**Obs**	**RR**	**Obs**	**RR**	**95%**	**CI**	**Obs**	**RR**			**Obs**	**RR**			**Total**	***P*-trend**
*Anal cancer*																
*Age*																
< 40	10	1.00	58	1.31	0.88	1.97	26	1.73	1.12	2.69	16	3.27	2.04	5.26	110	<0.001
⩾40	80	1.00	225	1.11	0.91	1.34	70	0.96	0.75	1.22	24	0.87	0.62	1.23	399	0.460
																
*Cervical cancer*																
*Age*																
< 40	614	1.00	2816	1.04	0.97	1.11	1014	1.14	1.06	1.24	324	1.16	1.05	1.29	4768	<0.001
⩾40	375	1.00	938	0.88	0.78	1.01	372	0.96	0.82	1.12	137	0.94	0.76	1.17	1822	0.791

a Estimated by Poisson regression models including terms for age, sex, period, county and socioeconomic index.

b Reference category.
